# Defining severe asthma – an approach to find new therapies

**DOI:** 10.3402/ecrj.v1.24356

**Published:** 2014-06-05

**Authors:** Alexandra M. Nanzer, Andrew Menzies-Gow

**Affiliations:** Asthma and Allergy, Royal Brompton & Harfield NHS Foundation Trust, London, United Kingdom

**Keywords:** severe asthma, comorbidities, phenotypes, new biological treatments

## Abstract

Asthma is a chronic inflammatory disease that has reached epidemic proportions worldwide. It is treatable in the majority of patients, but there is no cure. Moreover, a proportion of patients suffer from severe, difficult-to-control disease with daily symptoms and high morbidity, making it imperative that we continue to improve our understanding of the underlying mechanisms of this disease. Severe asthma is a heterogeneous condition. A systematic approach to identify specific asthma phenotypes, including clinical characteristics and inflammatory processes, is the first step toward individualized, logical therapy. This review focuses on the need to characterize severe asthma phenotypes and on novel, targeted molecular treatment options currently under development.

Asthma is associated with significant morbidity and mortality and is one of the most common long-term conditions worldwide (1). The prevalence of asthma has been rising over the past decade and currently affects up to 10% of the population in developed countries (2, 3). Fortunately, a majority of patients achieve control if they take their medication regularly and as prescribed, and international guidelines assist the treating physician to implement a stepwise escalation of treatment until symptoms are controlled (2). However, a substantial number of patients fail to gain clinical benefit from any of the standard treatments. Poor compliance may be a reason for this. However, it is noteworthy that studies assessing asthma control observed persistent symptoms in over 20% of patients despite treatment adherence in exceptional trial circumstances and in selected patient groups (3). It is therefore widely acknowledged that a small proportion of patients have a genuine poor response, in particular to corticosteroids. These patients have a greater risk of dying of their disease and their lives are often blighted by their condition, notwithstanding the disproportional amount of healthcare resources spent.

A well-defined, unbiased approach that defines the clinical characteristics that specify distinct phenotypes of asthma is critical for a better understanding of disease pathogenesis and thus successful management.

It is essential to differentiate severe asthma from difficult-to-control asthma. Assessment of each and every patient presenting with recurrent episodes of wheezing, chest tightness, and/or cough requires a careful history focusing on symptoms, exacerbating triggers and occupational as well as environmental factors. With the correct diagnosis established, systematic evaluation has the potential to identify up to half of previously deemed severe asthmatics as difficult-to-treat asthmatics (4, 5). Patients can be labeled as suffering from severe asthma with ongoing symptoms after an alternative diagnosis has been excluded, comorbidities have been treated, allergen exposures are being avoided and, last but not least, adherence with treatment and inhaler technique has been checked (Fig. 1).

**Fig. 1:**
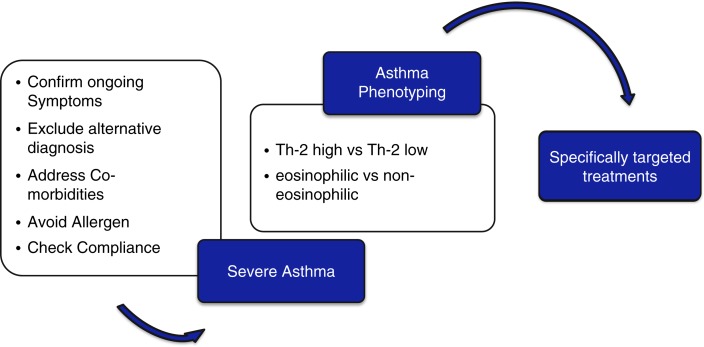
Investigation and treatment of severe asthma. A stepwise approach toward individualized asthma treatment incorporates a systematic assessment with a critical review of diagnosis, comorbidities and compliance.

In 2013, the international European Respiratory Society–American Thoracic Society guidelines classified severe asthma as a disease that requires treatment with high-dose inhaled corticosteroids and/or systemic corticosteroids to prevent it from becoming uncontrolled or that remains uncontrolled despite this therapy (6). Any of the following criteria qualifies a patient as having ongoing symptoms and therefore severe asthma:

1. Consistently poor symptom control: Asthma Control Questionnaire (ACQ) score is consistently >1.5 or Asthma Control Test (ACT) score is <20.
2. Frequent severe exacerbations requiring at least courses of oral corticosteroids for a minimum of 3 days in the previous year.
3. One or more hospitalization, intensive care unit admission, or invasive ventilation in the previous year.
4. Forced expiratory volume (FEV_1_)<80% predicted (with FEV_1_/FVC (forced vital capacity) reduced to less than the lower limit of normal).

## Comorbidities

Atopic rhinosinusitis, gastroesophageal reflux disease (GERD), sleep apnea, and obesity are well-known comorbidities that can aggravate and perpetuate and/or masquerade as asthma. Epidemiological studies report less atopy by skin test in severe asthma, but a high prevalence of chronic rhinosinusitis and nasal polyposis (7, 8). Severe asthma is disproportionally high in aspirin-exacerbated respiratory disease (AERD), where the majority of patients suffer from nasal polyposis (9).

Symptoms of GERD include cough and chest tightness. Heartburn and indigestion contribute to night awakenings and may mimic asthma. Prevalence of GERD in asthma is variable and can range from 12 to 85% (10). Treatment has proven beneficial for asthma-related quality of life. It was shown to reduce nocturnal symptoms and exacerbation frequency, and some studies report reduction in the use of short-acting beta_2_ agonists (11). Notwithstanding this, bronchodilators can potentially aggravate GERD by reducing esophageal sphincter tone (12).

Obesity is a major risk factor for asthma, and the epidemic increase in obesity is likely to be a significant contributor to the increase in numbers of patients with severe asthma. Factors such as insulin resistance, altered adaptive and innate immunity, changes in mechanical loading of the chest wall and abdomen, and increased airway hyperresponsiveness secondary to low-lung-volume breathing have all been linked to obesity-related asthma (13). Weight loss has been shown to improve asthma control, and a recent paper reported reversal of a proinflammatory cytokine milieu after bariatric surgery (14, 15). Treatment of obesity-related asthma with corticosteroids often proves disappointing. The pivotal role of adipose tissue resident immune cells (i.e. macrophages and mast cells as well as proinflammatory adipocytokines) makes them targets for novel biological treatments such as peroxisome proliferator activated receptor (PPAR) agonists and, in particular, in association with sputum eosinophilia, anti-interleukin (IL)-5 (16, 17).

Severe asthma is frequently associated with fungal and/or mold sensitivity. Patients with persistently uncontrolled asthma are often chronically colonized with *Aspergillus*, *Candida*, *Penicillium*, and *Curvularia* species (18–20). All patients should undergo both skin prick testing (SPT) and specific serum IgE tests: SPT has a sensitivity of around 50–60%, less than half of patients have a positive reaction to both tests and there is generally insufficient concordance between the two tests (21–23).

The first case reports linking asthma and obstructive sleep apnea (OSA) emerged in the late 1970s (24). Snoring, observed apnea, and poorly controlled asthma are closely linked, and patients who have OSA and nocturnal asthma may have similar clinical presentations (25–28). Treatment with continuous positive airway pressure (CPAP) significantly improves asthma quality of life, lung function, and short-term beta_2_ agonist requirements (29, 30).

Anxiety and depression are subjective emotions that may escape the attention of clinicians *and* patients with chronic diseases. Patients with severe asthma are under protracted distress and are at increased risk of developing psychiatric disorders, most commonly panic disorder, depression, and anxiety (31, 32). Moreover, studies have shown decreased asthma control with higher exacerbation rates in patients suffering from anxiety with or without depression (33, 34). The challenges of understanding and responding appropriately to the emotional factors of the chronic disease that asthma is mustn't be neglected, and patients might benefit from formal psychological support services.

Smoking is as common in asthmatics as it is in the general population (35, 36). Smoking asthmatics have a more rapid decline in lung function, more symptoms and exacerbations than nonsmokers and an impaired steroid response (37). Their inflammatory profile in blood and sputum differs compared to that of nonsmoking asthmatics (38). All patients should be offered smoking cessation advice (38–40).

## Asthma phenotyping

Recently, a significant effort has been directed at defining severe asthma and in particular its subgroups or phenotypes. Asthma is a heterogeneous disease, and phenotype-specific therapies promise enhanced treatment success. A phenotype is defined as the apparent characteristics of an organism resulting from its interactions with the environment and its genetic makeup (41).

**Fig. 2:**
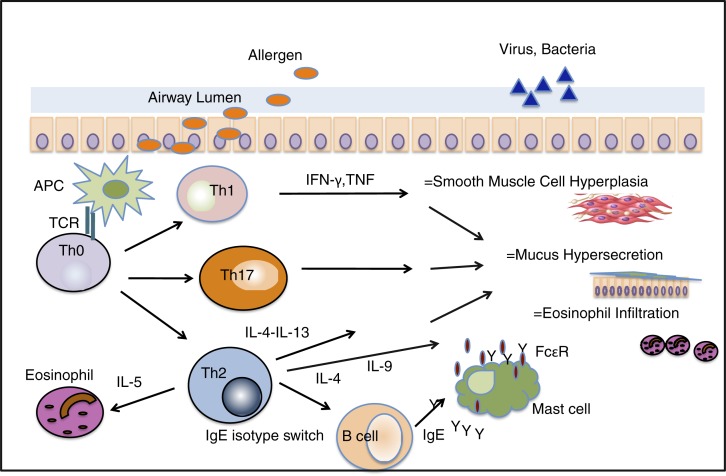
Immunopathology in asthma. Allergens are presented to naïve T-helper cells (Th0) via antigen presenting cells (APCs), resulting in the differentiation into Th1, Th17 and Th2 cells and the release of cell-specific cytokines. Th2 cytokines mediate airway eosinophil and mast cell recruitment, B-cell IgE isotype class switching, and mucus secretion. Allergen specific B-cells switch from IgM-producing to IgE-producing cells. Interleukin-17, which is produced by Th17 cells, mediates airway neutrophilia by inducing the production of CXC chemokine. IL=interleukin, TCR=T-cell receptor, IFN-γ=interferon gamma, TNF=tumor necrosis factor, FcɛR=high-affinity IgE receptor.

Historically, asthma has been termed a T-helper cell type 2 (Th2)-driven disease characterized by reversible airway obstruction, thickened airway smooth muscle cells, subepithelial fibrosis, and a characteristic aberrant immune regulation with a predominance of Th2 cells secreting cytokines IL-4, IL-5, and IL-13 (42). IL-4 is a key element in driving differentiation from naïve Th0 cells to Th2 cells, and it promotes B cell class switching to IgE production, mast cell growth, and eosinophil recruitment (43). IL-5 is responsible for driving eosinophil differentiation, survival and tissue cytotoxicity (44), and IL-13 mediates airway hyperactivity and increased mucus production and also promotes B cell IgE production. Other cells known to be central to allergic inflammation are mast cells, eosinophils, neutrophils, macrophages, dendritic cells and, in recent years, invariant natural killer T cells, innate lymphoid cells, and Th17 cells (Fig. 2).

Several approaches have been taken to characterize asthma subgroups. The Severe Asthma Research Program (SARP) identified five asthma subphenotypes by unbiased cluster analysis, three of which are severe asthma (45): the early-onset allergic type and the late-onset eosinophilic phenotype are both orchestrated by Th2 cells. They are clinically distinct yet overlap immunologically. A Th2-cell signature is further believed to play a predominant role in exercise-induced asthma (EIA), with mast cells and their mediators understood to be driving inflammation in EIA (46) and AERD. A lack of Th2 biomarkers is seen in obesity-related asthma and neutrophilic asthma (see Table 1).

**Table 1:** Asthma phenotypes according to their cytokine profiles divided into Th2-high and Th2-low asthma (41)

T cell signature	Phenotype	Biomarkers	Therapy
Th2-high	Early-onset allergic	Specific IgE, +SPT	Corticosteroids, anti-IgE
	Late-onset eosinophilic	Sputum eosinophilia, IL-5	Poor response to corticosteroids, anti-IL-5
	Exercise induced	Mast cells	Leukotriene receptor antagonists, SABA
Th2-low	Obesity related	Mast cells, adiponectin, Th1 cytokines	Poor response to corticosteroids Weight loss, PPAR agonists
	Neutrophilic	Th17, sputum neutrophilia	Vitamin D, p38 MAPK inhibitors, Macrolides. Poor response to corticosteroids

In another study using cluster analysis, sputum eosinophilia was incorporated. Consequent upon this, a phenotype of early-onset severe atopic asthma, late-onset asthma with persistent eosinophilia, and a cluster of obese females with late-onset asthma without eosinophilia were identified (47).

Molecular phenotyping has led to the development of biomarkers that specifically target Th2 responses in the lung: Woodruff and colleagues identified periostin (*POSTN*), chloride channel regulator 1 (*CLCA1*), and serpin peptidase inhibitor, clade B, member 2 (*SERPINB2*) as epithelial genes that were specifically induced in asthma and directly regulated by IL-13 *in vitro* (48). They further identified patients with distinctly higher levels of IL-5 and IL-13, termed Th2-high asthma. This was in contrast with patients with cytokine expression similar to that of healthy controls, including Th1 cytokines IL-12 and interferon gamma (IFNγ), which were significantly lower in the Th2-high group. The Th2-high and Th2-low groups also differed clinically, with the Th2-high group showing significant higher atopy and higher eosinophil levels in peripheral blood and bronchoalveolar lavage (BAL). Of particular interest was their observation of corticosteroid responsiveness: lung function of Th2-low asthmatics failed to improve following treatment with inhaled corticosteroids; in fact, patients’ FEV_1_ deteriorated in the first month, suggesting a detrimental effect of corticosteroids in Th2-low asthma (48).

## Novel therapies – targeting the right patient: Th2-high asthma

Omalizumab remains so far the most successfully applied monoclonal antibody to treat allergic Th2-high asthma by reducing the exacerbation rate. Omalizumab is a humanized IgG1k monoclonal antibody that specifically binds to free human immunoglobulin E (IgE), but not to IgE that is already bound by the high-affinity IgE receptor (FcɛRI) on the surface of mast cells, basophils, and antigen-presenting dendritic cells. Steric hindrance by the receptor means the receptor is not accessible to omalizumab binding, thus averting anaphylaxis. In a number of studies conducted so far, patients treated with omalizumab reported significant improvements in asthma-related symptoms, allowing for a reduction in corticosteroid and rescue inhaler use (49–51). It appears that patients with blood eosinophilia, high levels of exhaled nitric oxide, and serum periostin most benefit from anti-IgE treatment (52). However, evidence is now emerging that omalizumab has a role in non-atopic asthma. A recent trial demonstrated significantly increased asthma control in 29 non-atopic patients and a trend to reduced exacerbation rates and improved lung function (53, 54). A small randomized controlled trial comparing treatment with omalizumab and placebo in non-atopic asthmatics over a period of 16 weeks found a trend toward a decrease in exacerbations and a significant improvement in lung function in omalizumab-treated patients, as compared with the placebo group. In addition, the authors showed that the expression of the high-affinity IgE receptor on blood plasmacytoid dendritic cells decreased significantly in the active group but not in the placebo group (54). Larger trials, and in particular trials shedding light on how best to identify non-atopic patients who are likely going to respond to anti-IgE treatment, are needed.

The success of anti-IgE therapy is limited by the fact that IgE production is not affected. Treatment has to be given regularly and on a long-term basis. Brightbill et al. have successfully created a monoclonal antibody against the M1′ segment of membrane IgE on human IgE-switched B cells, resulting in depletion of IgE-switched B cells via apoptosis or/and antibody-dependent cell-mediated cytotoxicity (55). Total IgE levels were reduced without other immunoglobulin isotypes being affected. A phase IIb randomized controlled trial is currently testing its efficacy in uncontrolled allergic asthma, and study results are expected in 2015 (http://clinicaltrial.gov/ct2/show/NCT01582503).

Promising newer treatments targeting the Th2 pathway with steroid-sparing potential include the anti-IL-5 antibody mepolizumab. The first randomized controlled trials with anti-IL-5 were conducted in patients with mild to moderate asthma (56, 57). These studies failed to show a beneficial effect on lung function. However, after targeting patients with severe asthma and refractory airway eosinophilia and choosing the correct primary outcome (i.e. asthma exacerbations), anti-IL-5 treatment has been shown to significantly reduce exacerbations and oral corticosteroid doses required to control symptoms, and it has been well tolerated during the study period of over a year (58). Benralizumab, an IL-5 receptor alpha subunit (IL-5Rα) antibody, not only reduced peripheral blood eosinophils but significantly reduced eosinophil counts in airway mucosa and submucosa (59). However, neither of the two treatments has had any effect on lung function or patient-rated asthma control.

Clinical trials have also investigated targeted therapies against the Th2 cytokines IL-4 and IL-13. Pitrakinra is an IL-4 mutein, which binds to the IL-4Rα subunit and prevents the inflammation induced by IL-4 and IL-13. It has been shown to reduce allergen-induced airway responses when given in inhaled or subcutaneous form in a study of mild asthmatics and to reduce exacerbation rates in those with eosinophilia (60, 61). In moderate to severe asthma, the fully human monoclonal IL-4R-α dupilumab improved lung function, symptoms and exacerbation rate (62).

Periostin has proven to be a prognostic biomarker for treatment with the anti-IL-13 antibody Lebrikizumab. A recent study demonstrated that treatment with Lebrikizumab increased FEV_1_ in patients with a high serum periostin level (63). To date, the most successful anti-Th2 cytokine therapies have focused on accurate identification of a patient's phenotype to allow for personalized treatment regimes.

## Non-Th2-high asthma: a different challenge

Treatments targeting other possible inflammatory mediators have shown less clear evidence of clinical benefit, and the reason for this might stem from the difficulty of a clear definition: Th2-low asthma remains identified by the absence of Th2 biomarkers. Although the presence of a neutrophilic phenotype of asthma has been suggested, its use as a biomarker is imperfect. In contrast to the significant association between blood eosinophilia and airway eosinophilia in patients with asthma (who are not treated with systemic corticosteroids), there is no correlation at all between blood neutrophilia and airway neutrophilia (64). Furthermore, there is no consent as to the level of airway neutrophilia that would define pathology; neutrophils, unlike eosinophils, are a normal component of the cells retrieved in induced sputum. There are robust data, however, that the later onset, obese, non-eosinophilic phenotype is often particularly steroid insensitive and difficult to control. Interestingly, patients with severe asthma are found to have higher levels of the pro-inflammatory cytokine IL-17A in sputum and BAL, and the severity of airway hypersensitivity correlates with airway neutrophilia and levels of IL-17A expression (65–67). Studies in mice and humans suggest an association between steroid resistance and Th17-mediated disease: adoptive transfer of Th17 cells resulted in increased levels of CXC chemokines and G-CSF in the BAL fluid of SCID mice, and treatment with dexamethasone resulted in increased neutrophil numbers but no improvement of airway hyperresponsiveness (68). Glucocorticoid resistance is associated with an increased expression of the transcriptionally inactive glucocorticoid receptor beta (GR-β) (69–71). Furthermore, GR-β increases in response to exogenous IL-17A and IL-17F, an effect that was more prominent in asthmatics than in healthy controls (72). Further accounting for corticosteroid insensitivity are defects in glucocorticoid receptor binding and activation of transcription factors such as AP1, which is activated by pro-inflammatory cytokines such as tumor necrosis factor alpha (TNFα) or through failure to induce regulatory cytokines like IL-10 (73).

Anti-TNFα treatment was tested in severe (etanercept) (74, 75) and moderate (infliximab) (76) asthmatics and resulted in improved asthma control, FEV_1_, and bronchial hyperreactivity. However, the effect ceased as soon as the drug was discontinued. The fully human anti-TNF antibody golimumab did not have any clinical benefit, but resulted in an increase of respiratory infections and malignancies leading to an early discontinuation of the trial (77).

In a randomized controlled trial, the human anti-IL-17 receptor monoclonal antibody brodalumab was not superior to placebo in terms of asthma control (78). Interestingly, the authors observed a nominal significance in a subgroup of patients with high bronchodilator reversibility. Further studies of brodalumab in this asthma subpopulation are warranted. Anti-IL-9 (79), agents targeting TSLP (80), chemokine inhibitors (CCR3 and CCR4) (81), phosphodiesterase and kinase inhibitors (82) and the use of vaccination (83) with the aim to shift from Th2 to Th1 are all under development, but have yet to show any clear clinical benefit.

Deficiency in serum vitamin D has been linked to predisposing individuals to chronic inflammatory lung disease, such as asthma and viral respiratory infections, with higher rates of hospital admission for respiratory diseases (84–86). Reports have shown a positive correlation between vitamin D deficiency and asthma prevalence (87–89). Manipulation of vitamin D status for therapeutic benefit in asthma is currently highly topical, with studies suggestive of a role in steroid-refractory asthma (90, 91).

Macrolide antibiotics have anti-inflammatory and immune-modulatory effects. They also increase gastrointestinal motility and to that extent might prove beneficial in patients with significant GERD. They have been proven effective in chronic respiratory diseases such as cystic fibrosis (CF) and chronic obstructive pulmonary disease (COPD). They have been shown to reduce severe exacerbations in patients with non-eosinophilic asthma and to have significantly improved the Asthma Quality of Life Questionnaire score (92). However, chronic antimicrobial use is associated with the risks of population resistance, and treatment should be restricted to severe asthma patients at greatest unmet need despite optimal asthma management.

Methotrexate has proven a valid agent in patients who, despite long-term treatment with oral glucocorticosteroids, fail to gain satisfactory control of their asthma (93). Due to considerable side effects, close monitoring is needed and treatment should only be initiated in specialist centers.

## Bronchial thermoplasty

Smooth muscle hyperplasia is a distinctive feature of asthma. Applying radiofrequency energy to subsegmental airways has been shown to reduce muscle mass at the site of thermoplasty. Trials demonstrated a reduction in the number of severe asthma exacerbations and an improvement in asthma-specific quality of life (94), and a recent study found no adverse events after a 5-year follow-up period (95). Although guidelines recommend bronchial thermoplasty for adults with severe asthma that is not controlled with inhaled corticosteroids and long-acting beta_2_ agonists (LABAs), it is currently unclear which phenotypes respond best to the treatment. Studies are needed to identify the phenotype of patients who will derive significant clinical benefit most from this invasive procedure.

## Conclusion

Asthma is common, and in most cases it is a treatable disease. However, patients with difficult-to-treat asthma remain a challenge for every healthcare professional, and there is a significant unmet clinical need. It has become abundantly clear that asthma is a heterogeneous disease in its course and variation in response to treatment. A systematic approach to the complexity and diversity of asthma pathophysiology is essential. Up until recently, trials testing anti-inflammatory therapy based on targeting cytokines have, despite highly promising results in animal models, proven to be disappointing. However, in carefully selected patients, when asthma phenotypes are taken into account, novel biological treatments may lead to significant advances. Discriminatory biomarkers and genetic profiling may aid identification in support of personalized pharmacotherapy. Within the next 5 to 10 years, logical, targeted therapies will be available for patients with Th2-high severe asthma, and our understanding of the mechanisms driving Th2-low asthma will be significantly advanced.
